# Using a Carbon Quantum Dot Suspension as a New Solvent for Clear Hydrophobic Surface Coating on Hydrophilic PVA Films

**DOI:** 10.3390/polym16172513

**Published:** 2024-09-04

**Authors:** Yena Oh, Kitae Park, Jamilur R. Ansari, Jongchul Seo

**Affiliations:** Department of Packaging and Logistics, Yonsei University, 1 Yonseidae-gil, Wonju-si 26493, Republic of Korea; ooohyena_01@yonsei.ac.kr (Y.O.); rbdnjs0504@yonsei.ac.kr (K.P.); jransari.phd@gmail.com (J.R.A.)

**Keywords:** high-barrier packaging, hydrophobic coating, carbon quantum dot, morphology, polyvinyl alcohol

## Abstract

Polyvinyl alcohol (PVA) is a popular material used in the packaging industry. However, it is vulnerable to moisture, which can affect its performance and durability. Introducing hydrophobic substances, such as tetraethyl orthosilicate (TEOS) and hexadecyltrimethoxysilane (HDTMS), on the top layer of PVA can help maintain the excellent properties of PVA under high-humidity conditions. The low compatibility of hydrophobic materials with the hydrophilic layers allows them to aggregate more easily. To overcome these issues, we focused on the effects of particle size when increasing the coating suspension’s dispersibility. A carbon quantum dot (CQD) suspension is an appropriate novel solvent for hydrophobic TEOS/HDTMS coating suspensions because its particles are small and light and exhibit good dispersibility. The CQD suspension formed a smooth hydrophobic coating on the TEOS/HDTMS materials. Furthermore, the uniformly coated PVA with the CQD suspension exhibited a water contact angle of 110°. The water droplets remained intact without being absorbed, confirming the effectiveness of the surface coating facilitated by CQDs. These results suggested that CQDs improved the dispersibility and enhanced the coating quality of TEOS/HDTMS on PVA. Enhancing the hydrophobicity of PVA is ideal for applications in packaging and other fields.

## 1. Introduction

The increased use of polymeric products, especially in packaging, highlights the urgent need to develop biodegradable polymer films to reduce the environmental impact [1]. Among various biodegradable polymers, polyvinyl alcohol (PVA) has attracted considerable attention for packaging applications because of its high transparency and oxygen barrier properties [2,3,4,5,6]. However, the intrinsically hydrophilic properties of PVA caused by the hydroxyl groups in repeating units result in low oxygen barrier properties under high-humidity conditions and water vulnerability, substantially limiting its practical application in the packaging of water-based products [7,8,9]. Researchers have explored strategies, such as chemical and physical crosslinking reactions, to overcome these limitations [8,9,10,11]. However, the hydrophilicity of PVA is not completely modified by the crosslinking reaction, and the crosslinked PVA still exhibits low water resistance. Therefore, the introduction of hydrophobic coating materials such as tetraethyl orthosilicate (TEOS) and hexadecyltrimethoxysilane (HDTMS) on the top layer of PVA can help maintain its properties under high-humidity conditions.

Spray coating is commonly used for multilayered films because of the ease of thickness control and its ability to coat large surfaces [12]. However, hydrophobic coating materials can aggregate because of the low dispersibility of the TEOS/HDTMS coating suspension and the weak interaction of the coating with hydrophilic substrates during the spray coating process [13]. While increased surface roughness due to aggregation can enhance hydrophobicity, it can also detract from the superior appearance qualities of PVA and hinder the homogeneous surface characteristics desired in packaging materials [14]. High dispersibility in the hydrophobic coating suspension can ensure uniform coating. Therefore, it is essential to consider the interactions among the components of the coating suspension. The sizes of the components in the coating suspension can influence various properties [15], thereby affecting the dispersion of the coating suspension. In addition to the surface chemistry, physical properties such as particle size also affect dispersibility [16]. The effect of particle size on dispersibility is a topic of research in many industries, such as technology, food, and medicine. Liu et al. found that plant-based protein particles in an ice cream showed higher dispersibility with smaller sizes, with particles smaller than 1 μm exhibiting the highest dispersibility and 145 μm particles the lowest [15]. Xu et al. reported that smaller antimony nanoparticles exhibited a slower sedimentation velocity and improved lubricant dispersion stability [17]. These examples illustrate the relationship between particle size and dispersibility.

Carbon quantum dots (CQDs), which are unique carbon nanomaterials with sizes of less than 10 nm, can be easily synthesized from various sources, making them highly accessible [18,19,20,21]. In particular, their attractive features, such as chemical inertness, UV blocking, and antimicrobial properties, have led to their emergence as fillers for packaging applications [22]. Furthermore, owing to the excellent dispersion stability of CQD particles caused by their extremely small size and lightweight nanoparticles, CQD suspension may be suitable as a novel solvent for hydrophobic TEOS/HDTMS coating [22]. To the best of our knowledge, no studies have reported the manifestation of novel properties or the use of CQD suspension as a solvent. To explore the feasibility of using a CQD suspension as a novel solvent for hydrophobic TEOS/HDTMS on a hydrophilic PVA film, in this study, the effect of CQD nanoparticles on enhancing the dispersibility of TEOS/HDTMS coatings was investigated. The CQD suspension was prepared using green tea extract (GTE), so GTE can be considered a precursor stage before the particle size becomes as fine as that in the CQD suspension. Therefore, GTE served as a comparative group for solvent particle size along with H_2_O. Given that H_2_O, GTE, and CQD suspensions have different particle sizes, their dispersibility behaviors were analyzed accordingly. Furthermore, using three different solvents (H_2_O, GTE, and CQD suspension), a series of PVA films coated with TEOS/HDTMS were prepared. The chemical structure, morphology, surface hydrophobicity, optical, and oxygen barrier properties of the films were characterized.

## 2. Materials and Methods

### 2.1. Materials

Commercial-grade (F-17 grade) PVA resins (degree of hydrolysis: 98.0–99.5%, molecular weight: 74,800 g/mol) were provided by OCI Co., Ltd. (Incheon, Republic of Korea). Boric acid (BA, purity: 99%), hydrogen chloride (assay: 35%, HCl), TEOS (assay: 98%), and HDTMS (assay ≥ 85%) were procured from Sigma-Aldrich Chemical Co. (St. Louis, MO, USA). Green tea was purchased from Bosung Jeda Co. (Bosung, Republic of Korea). Deionized water was used in all experiments.

### 2.2. Sample Preparation

#### 2.2.1. Preparation of Crosslinked-PVA (CPVA, CP) Films

First, PVA (10 g) was dissolved in 100 mL of water and stirred at 90 °C for 3 h. BA (0.5 g) was dissolved in 25 mL water and stirred at 40 °C for 10 min. Subsequently, the BA solution was added to the PVA solution and stirred at 90 °C for 2 h. The PVA and BA mixture was cooled to 50 °C, then 0.4 mL of HCl was added. This solution was applied onto a glass substrate using a bar coater and subsequently dried in an oven at 25 °C for 12 h. After peeling the crosslinked PVA film from the glass substrate, characterization tests were performed.

#### 2.2.2. Film Coating

Before preparing the TEOS/HDTMS suspensions, the GTE and CQD suspensions were prepared for incorporation into the coating solution. Green tea leaves (5 g) were crushed and stirred into 100 mL of water at 80 °C for 1 h to obtain the GTE. To synthesize the CQDs, green tea leaves (5 g) were sonicated in 100 mL of water for 10 min. Subsequently, they were stirred for 30 min and hydrothermally treated at 200 °C for 5 h. After cooling the mixture to 25 °C, it was filtered through a Whatman filter paper. Subsequently, the mixture was centrifuged at 14,000 rpm for 15 min to isolate the CQD suspension.

The TEOS/HDTMS suspensions with different solvents were prepared individually. Ethanol (36.5 mL) and H_2_O, GTE, or CQD solution (8 mL) were used as solvents. They were stirred and mixed for 10 min, followed by the addition of TEOS (4 mL) and HDTMS (1.5 mL), and stirred for another 30 min. Finally, HCl (0.01 mL) was added and the mixture was stirred for an additional 10 min to complete the coating suspension. The coating suspensions were prepared at 25 °C. The TEOS/HDTMS suspensions were placed in spray bottles and sprayed onto the PVA films ten times. Subsequently, the TEOS/HDTMS-coated films were dried at 25 °C for 12 h.

### 2.3. Characterization

The morphology and high-resolution transmission electron microscopy (HRTEM) images of the CQDs were obtained using a JEM-ARM200F (NEOARM) instrument (JEOL Ltd., Tokyo, Japan). The TEM images were analyzed using the image processing software ImageJ 1.54, and the lattice fringes were calculated. The chemical structures of the samples were analyzed using Fourier transform infrared (FTIR) spectroscopy using a Spectrum 65 FTIR spectrometer (Perkin Elmer Co., Ltd., Waltham, MA, USA). Spectra were recorded from 4000 to 400 cm^−1^ in attenuated total reflection (ATR) mode at a scan speed of 64. Micrographs were obtained with scanning electron microscopy (SEM) at 500× magnification using a JEOL-7800F instrument (JEOL Ltd., Tokyo, Japan). Water contact angles (WCAs) were measured using a Phoenix 300 contact angle goniometer (SEO Co., Ltd., Suwon, Republic of Korea). UV-vis spectra were recorded in the 300–700 nm range using a UV-vis spectrophotometer (JASCO V-650; JASCO International Co., Ltd., Tokyo, Japan). Oxygen transmission rates (OTRs) were determined using an OTR 8001 oxygen permeability tester (Systech Instruments Co., Ltd., Johnsburg, IL, USA) following ASTM D3985 standards [23], and maintained at 23 °C and 0% relative humidity (RH).

## 3. Results and Discussion

**Figure 1:**
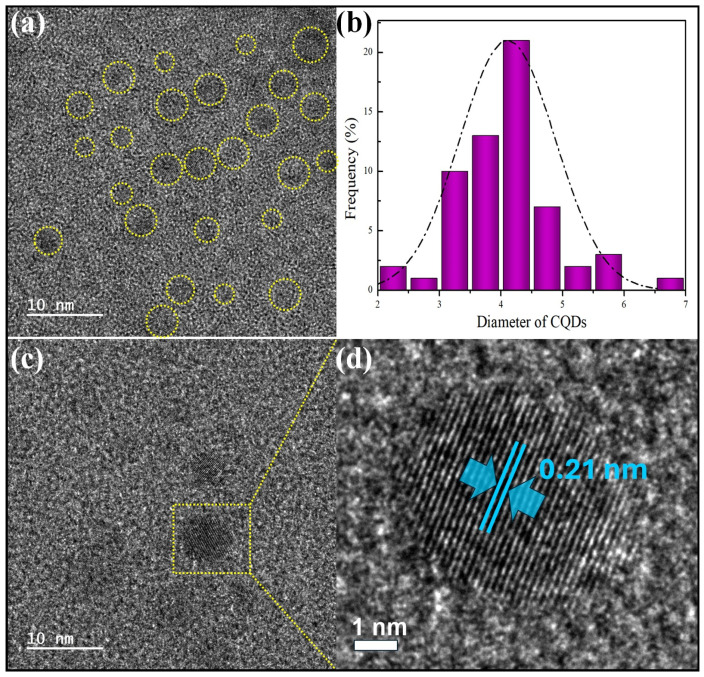
(**a**) TEM images, (**b**) histogram representing the particle size distribution, (**c**) high-resolution TEM (HRTEM) image, and (**d**) lattice spacing of the CQDs.

Before characterizing the film properties, a particle analysis of the co-solvent materials utilized as variables in the coating suspension was conducted. The morphology and size of the synthesized CQDs were determined by TEM. As shown in Figure 1a,c, the TEM images revealed that the CQDs were spherical, very small, and well dispersed. The particle size distribution of the synthesized CQDs ranged from 2.0 nm to 7.0 nm in diameter, showing a relatively narrow distribution. The average diameter was 4.1 nm ± 0.5 nm, which agreed well with the characteristic size of previously reported CQDs. Additionally, the HRTEM image revealed lattice fringes of 0.21 nm for the as-prepared CQDs, indicating the successful synthesis of the CQDs [24]. In contrast, Gopal et al. determined the particle size of GTE to be approximately 10–80 μm [25]. This suggests that the GTE particles were much larger than those of the CQDs. Therefore, in terms of particle size, the GTE particles could be considered for comparison with the CQD particles in the TEOS/HDTMS coating.

### 3.1. Dispersibility of the Coating Suspensions

**Figure 2:**
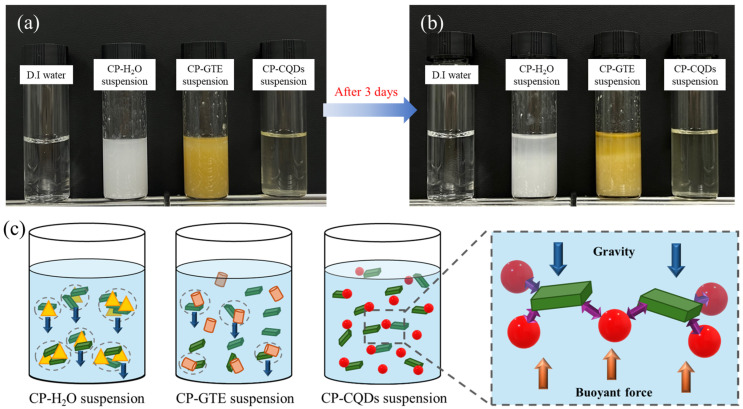
(**a**) TEOS/HDTMS suspensions with different solvents (H_2_O, GTE, CQDs), at the time of the dispersion in the solvents (0 h) and (**b**) after 3 d. (**c**) Theory for improving dispersion in the suspension.

The TEOS/HDTMS coating suspensions are depicted in Figure 2a,b. As shown in Figure 2a, the CP–H_2_O and CP–GTE suspensions were cloudy and opaque with the aggregation and slight precipitation of the TEOS and HDTMS. The degree of aggregation was more pronounced in the CP–H_2_O suspension. In contrast, the CP–CQD suspension became transparent, concurrent with a significant improvement in dispersion [26]. As the dispersion increases in a suspension containing nanoparticles, the suspension may become transparent. After 3 d, distinct phase separation occurred in the CP–H_2_O and CP–GTE suspensions, whereas the CP–CQD suspension remained well dispersed.

The theory behind the enhanced dispersion in the TEOS/HDTMS suspension with cosolvent changes is shown in Figure 2c. This was attributed to the extremely small and lightweight nature of the CQDs. The CQDs demonstrate excellent stability, as evidenced by the high zeta potential value (>30) reported in numerous studies [27,28]. The CQD particles can maintain a suitable equilibrium between buoyancy and gravity within the suspension [28]. It is estimated that the interactions and bonding between the dispersed CQD particles and TEOS and HDTMS contribute to the overall enhancement of dispersion within the suspension. For similar reasons, owing to the micro-sized particle characteristics of GTE, the CP–GTE suspension exhibited slightly improved aggregation and precipitation compared to the CP–H_2_O suspension. While the dispersion enhancement ability of the GTE is lower than that of the nano-sized CQDs, small particles can still improve dispersion, albeit to varying extents, depending on their size. Therefore, it can be inferred that the size and characteristics of the particles influenced their dispersion in the suspension.

### 3.2. FTIR Analysis

**Figure 3:**
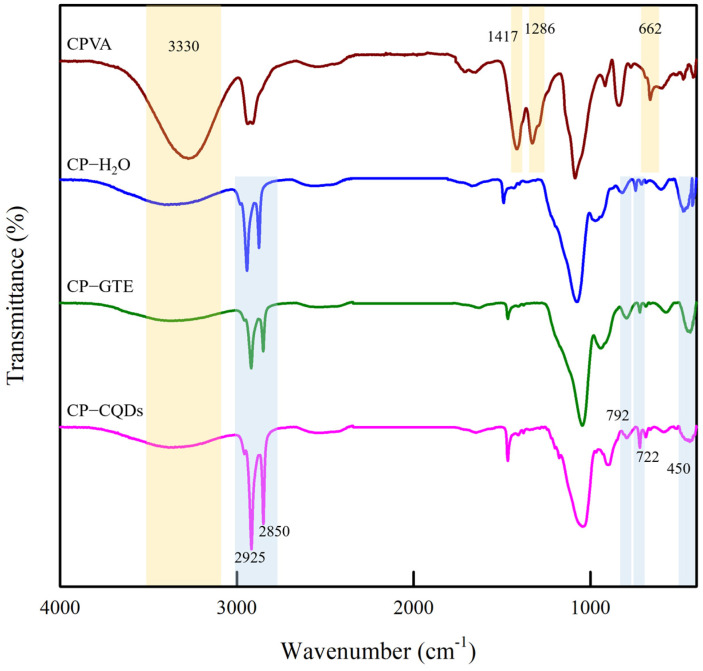
ATR-FTIR spectra of top surfaces of CPVA and CPVA coated with TEOS/HDTMS films.

The FTIR spectra of the CPVA and CPVA coated with TEOS/HDTMS are shown in Figure 3. FTIR analysis was conducted to examine the chemical bonding between the CPVA and TEOS/HDTMS coatings and investigate solvent-induced changes. The CPVA exhibited spectral peaks at 3330, 1655, 1422, and 1086 cm^−1^, corresponding to the –OH, C–O–C, and C–OH groups in the PVA chains, respectively [11,29,30]. In addition, peaks at 1417 (B–O), 1293 (B–O–C), 1030 (B–O–C), and 662 cm^−1^ (O–B–O) were detected because of the crosslinking reaction between the PVA and BA [10,11,31,32].

**Figure 4:**
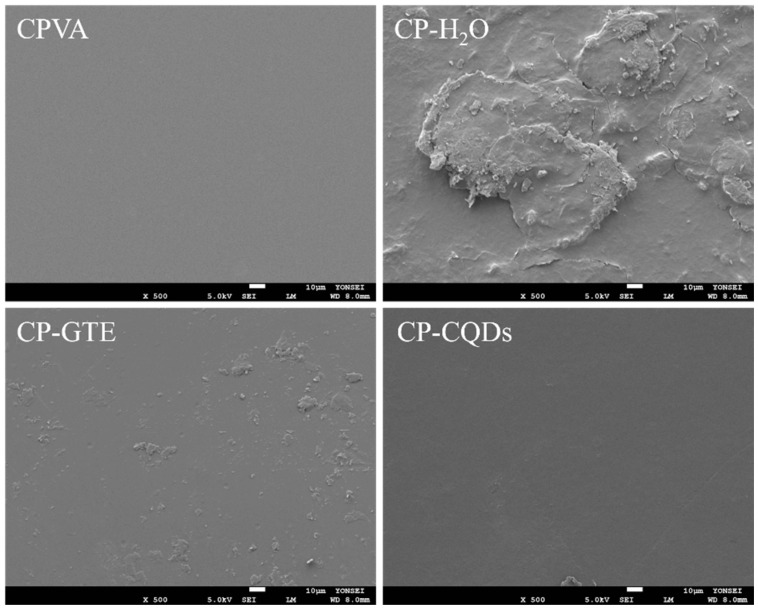
SEM images of the spray-coated TEOS/HDTMS surface with different solvents on the PVA films.

Upon coating CPVA with TEOS/HDTMS, a decrease in intensity at 3330 cm^−1^ was observed, with CP–CQDs showing the greatest decrease. Additionally, the intensities of the peaks at 1417, 1286, and 662 cm^−1^ gradually decreased and disappeared, while new peaks emerged after coating. The new peaks at 2925 and 2850 cm^−1^ corresponded to the stretching vibrations of -CH_3_ and -CH_2_, respectively, indicating long-chain hydrocarbons of the TEOS/HDTMS coating on the film surface [33,34,35]. Furthermore, the TEOS/HDTMS-coated films exhibited new peaks at 1056, 792, and 450 cm^−1^, corresponding to Si–O–Si bond vibrations, along with a peak at 722 cm^−1^ corresponding to Si–C [34,36,37,38]. The peak at 1470 cm^−1^ broadened as a result of overlapping C–O bonds [33,34]. The intensity of the newly emerged peaks was the weakest for CP–H_2_O and strongest for CP–CQDs. These changes suggested a decrease in the presence of PVA and BA on the surface and an increase in the presence of TEOS/HDTMS, which was attributed to the improved uniformity of the TEOS/HDTMS coating on the surface. Therefore, a more effective coating occurs when CQDs are used in the TEOS/HDTMS coating suspension. The differences in the coating forms depending on the composition are shown in Figure 4.

### 3.3. Film Morphology

Figure 4 shows the SEM images of the top surfaces of the CPVA film and TEOS/HDTMS spray-coated films with different solvents. The top surface of the CPVA film was uniformly smooth. In contrast, the top surface showed aggregation and large particles after TEOS/HDTMS spray coating with H_2_O. Particle counts and aggregations decreased when H_2_O was replaced with the GTE or CQD suspension, and CQDs worked better than the GTE particles. The key distinction between the GTE and CQDs lies in the particle size. Smaller particles contributed to enhanced dispersibility and a more uniform coating. These SEM results suggest that the relatively low compatibility between PVA and TEOS/HDTMS was successfully addressed by the excellent dispersibility of the particles.

### 3.4. Surface Hydrophobicity

**Figure 5:**
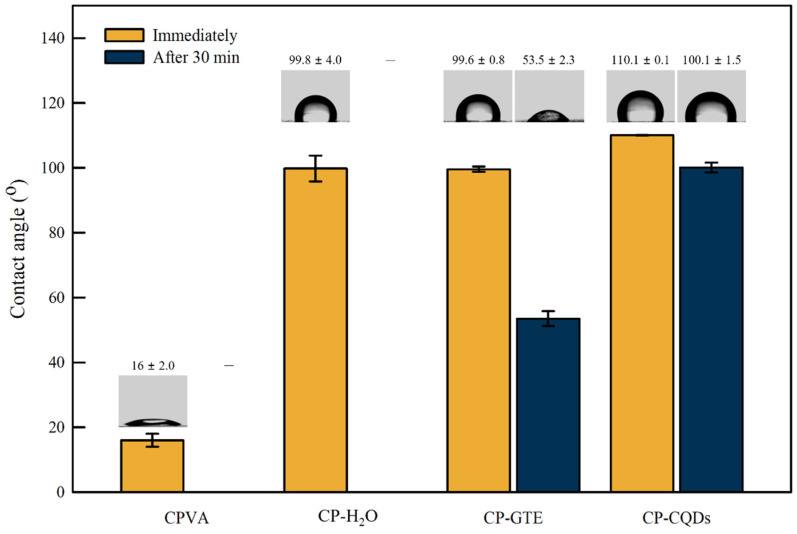
WCAs measured immediately and after 30 min for different TEOS/HDTMS coating suspensions.

Water contact angle (WCA) measurements were performed to investigate the hydrophobicity of both the CPVA and TEOS/HDTMS spray-coated films. As illustrated in Figure 5, measurements were taken immediately after the water droplet was placed on the surface and again after 30 min to observe any changes in the WCA. The CPVA film showed a WCA of 16°, whereas for the CP–CQDs film, the WCA significantly increased up to 110.1°. This indicates that the surface has become hydrophobic. The Si–O bonds on the coated surface contributed to this hydrophobicity, as described in FTIR. The WCAs of the CP–H_2_O and CP–GTE films were relatively small, with no significant differences observed between the two films. After 30 min, water penetrated and swelled the CPVA and CP–H_2_O films, rendering the WCA measurement impossible. For the CP–GTE and CP–CQD films, the degree of surface degradation was calculated as a percentage based on the changes in the WCA. CP–GTE and CP–CQD experienced surface degradations of 46.3% and 9.1%, respectively. The CP–CQD film exhibited the smallest change in the water contact angle, indicating virtually no deterioration.

Water can react with and be absorbed by the hydroxyl groups of the CPVA film. The FTIR data show that the TEOS/HDTMS spray-coated films formed Si–O groups on the surface and had fewer hydroxyl groups. It became hydrophobic because of these surface modifications. Among all the films, the CP–CQD film demonstrated the strongest and most stable hydrophobicity by maintaining the highest WCA for an extended period. This result is attributed to the uniform coating facilitated by the CQDs, as revealed by the SEM results (Figure 4) [39]. The homogeneous coating of the CP–CQD film reduced the influence of the hydroxyl groups, thereby enhancing the hydrophobicity and resistance to water penetration. Additionally, the CP–CQD film demonstrated the most consistent repeatability with minimal data scattering compared with the other films. This also indicates that CQDs alleviate aggregation and effectively promote coating quality when the TEOS/HDTMS spray coating is applied to the PVA surface [40,41].

### 3.5. Barrier Property

**Figure 6:**
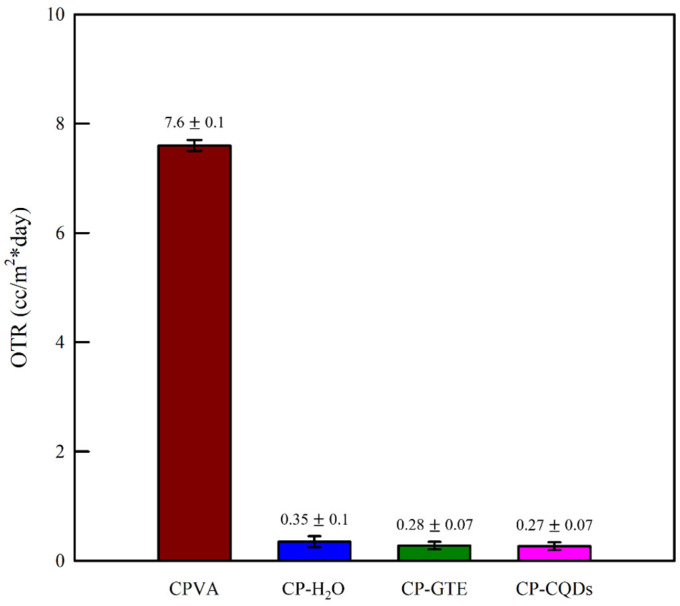
OTR values depending on the solvents used for the spray coating of TEOS/HDTMS.

The barrier properties of materials must be understood as they directly influence product quality and shelf-life maintenance. As shown in Figure 6, the OTR value of the CPVA film decreased significantly from 7.6 ± 0.1 cc/m^2^·day to 0.27 ± 0.07 cc/m^2^·day after applying the TEOS/HDTMS coating. This is because the SiOx coating formed by the combination of TEOS and HDTMS enhances the oxygen barrier properties owing to its dense structure and tortuosity effect [42,43]. Specifically, the CP–GTE and CP–CQD films showed a further decrease in the OTR and a relatively lower standard deviation, which could be related to the uniformity and compactness of the coated surfaces. In other words, the uniform coating layer on the CP–GTE and CP–CQD films, as shown in the SEM images (Figure 4), induced fewer voids or defects that could facilitate oxygen permeation.

### 3.6. Transparency

**Figure 7:**
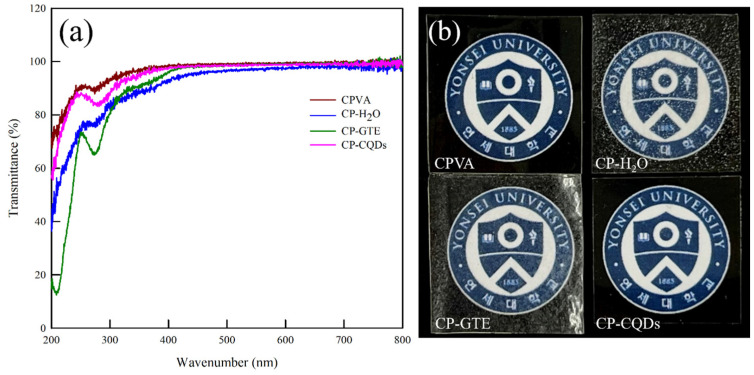
(**a**) UV-vis spectra and (**b**) photographic images showing the effect of various solvents used for the spray coating of TEOS/HDTMS.

Optical properties such as transparency are required for packaging applications. The transmittance of the coating was measured using UV-vis spectroscopy, as shown in Figure 7. Regardless of the solvent used, the transmittance of all samples was higher than 90% in the visible light region (400–800 nm). In addition, no discernible loss in the transparency of the CP–CQD film was observed in contrast to those of the CP–H_2_O and CP–GTE films. In general, factors influencing film transparency include its uniformity and aggregation. If a film lacks uniformity and exhibits aggregation, it can exhibit noticeable light scattering and/or absorption [44,45,46]. In other words, the high transparency of CP–CQDs is attributed to the homogeneous coating, whereas the decrease in the transparency of CP–H_2_O and CP–GTE is a consequence of the incompatibility between the hydrophobic coating suspension and the CPVA surface, resulting in aggregation. Additionally, a reduction in the transmittance of the CP–GTE spectrum was observed in the UV region (200–350 nm). This reduction is attributed to the UV protection properties of green tea ingredients, particularly polyphenolic families. The presence of these components contributed to the absorption of UV light, enhancing the overall UV-blocking capability of the CP–GTE film [47,48].

## 4. Conclusions

In this study, a CQD suspension was applied to address aggregation issues in the TEOS/HDTMS coating of the hydrophobic PVA surface. A TEOS/HDTMS coating layer was fabricated on the PVA surface using a spray coating method. The surface morphologies and physical properties of the PVA films coated with TEOS/HDTMS were strongly dependent on the solvent used. FTIR confirmed the formation of Si–O bonds by the reaction of TEOS/HDTMS and hydroxyl groups in PVA and a decrease in OH groups, indicative of the hydrophobic surface on the PVA films. This was demonstrated by the water contact angle increasing from 16° to 110.1°. In particular, when the CQD suspension was utilized as the solvent, as observed by SEM, surface aggregation was mitigated, significantly enhancing the coating uniformity. Moreover, an improvement in the coating surface morphology led to improved hydrophobicity, barrier properties, and transparency. These findings underscore the role of CQD suspensions in improving coating dispersibility and facilitating the formation of clean coatings. Thus, when endowing hydrophobicity to PVA surfaces via TEOS/HDTMS coating, the use of a CQD suspension enables the attainment of superior coating surfaces without aggregation issues, promising broader applications for PVA in the packaging industry. Further research is required to assess the safety and potential toxicity of this technology for packaging applications.

## Data Availability

Data are contained within the article.
